# Small endometrial carcinoma 10 mm or less in diameter: clinicopathologic and histogenetic study of 131 cases for early detection and treatment

**DOI:** 10.1002/cam4.139

**Published:** 2013-09-30

**Authors:** Katsuhiko Hasumi, Yuko Sugiyama, Kimihiko Sakamoto, Futoshi Akiyama

**Affiliations:** 1 Department of Gynecology, Cancer Institute HospitalTokyo, Japan; 2 Department of Pathology, Cancer InstituteTokyo, Japan

**Keywords:** Biological behavior, early diagnosis, early treatment, endometrial carcinoma, histogenesis, natural history, small carcinoma

## Abstract

Natural history and clinicopathologic features of early endometrial carcinoma are not evident. Its knowledge is essential to make up strategies for prevention, early detection, and treatment of endometrial carcinoma. Especially it is important to know pathways of endometrial carcinogenesis and frequency of endometrial carcinomas arising from endometrial hyperplasia. Clinicopathologically 131 patients with endometrial carcinoma measuring ≤10 mm in diameter (“small endometrial carcinoma”) were studied to get useful information for early diagnosis, treatment, and histogenesis. The entire endometrium of surgically removed uterus was step-cut and examined. The patients were, on average, 5 years younger than the controls whose carcinomas measure >10 mm (*P* < 0.0001). Of the 131 patients, 20% were asymptomatic although only 5% of the controls were asymptomatic (*P* < 0.0001). Seventy-six percent had the carcinomas located in the upper third section of the uterine corpus. Macroscopically 44% of the tumors were flat and 56% were elevated. Incidence of nodal and ovarian metastases were <1%. Forty percent of “small endometrial carcinomas” were associated with endometrial hyperplasia and 60% were not. It is logical to believe that there are two pathways of endometrial carcinogenesis: carcinomas occurring from hyperplasia (40%) and carcinomas occurring from normal endometrium (60%). As hyperplasia-carcinoma sequence is not a main route, we cannot probably prevent carcinomas only by treatment of hyperplasia. Effort must be focused on detecting early de novo carcinomas. As most “small endometrial carcinomas” arise in the upper third of the corpus, careful endometrial sampling there is important for early detection.

## Introduction

It is important to know pathways of endometrial carcinogenesis to make up strategies for prevention and early detection of endometrial carcinoma. There has been conception that two distinct mechanisms are responsible for onset of endometrial carcinoma: endometrial carcinoma occurring in complex hyperplasia and endometrial carcinoma occurring ab initio in nonhyperplastic endometrium (de novo carcinoma) [1, 2]. To investigate whether the conception is true, study of small-sized carcinomas and their adjacent noncancerous endometrium is essential. However, there has been no such report.

Clinically it is also important to know whether the hyperplasia-carcinoma sequence is a main route or a rare route. If the hyperplasia-carcinoma sequence were a main route, most of endometrial carcinomas could be prevented by early detection and treatment of hyperplasia [3]. However, as Silverberg stated the actual frequency of endometrial carcinoma arising from complex hyperplasia is not evident [4]. There has been no report free of biasing factors, which increase or decrease the actual frequency. The frequency may be overestimated if patients underwent unopposed estrogen replacement therapy [5]. The frequency may be underestimated if the endometrial carcinoma is large and replaces the antecedent hyperplasia. The frequency may be also underestimated if only few sections of the adjacent endometrium from each surgically removed uterus are prepared, as in such cases local endometrial hyperplasia may be missed. To investigate the actual frequency, study of small-sized endometrial carcinomas and their adjacent noncancerous endometrium free of the biasing factors mentioned above is essential.

It is generally accepted that endometrial carcinoma can be treated best by early diagnosis and early operation. However, how early should endometrial carcinoma be diagnosed for a perfect prognosis? Is there any reliable means to detect early endometrial carcinoma? Where does endometrial carcinoma commonly occur in the uterine corpus? What are the macroscopic features of early endometrial carcinomas? What are the symptoms of early endometrial carcinoma? What is the biological behavior of early endometrial carcinoma? What is the treatment of choice for control of early endometrial carcinoma? It is essential to study small-sized carcinomas for answering these questions.

One hundred and thirty-one patients having endometrial carcinomas with maximum dimension of 10 mm or less (“small endometrial carcinoma”) were studied clinicopathologically to get useful information for early diagnosis and treatment and to investigate whether hyperplasia-carcinoma sequence is a main route. Our present study is particularly valuable for the following reasons: (1) the entire endometrium of surgically removed uterus was step-cut and examined in all of the cases not to miss “small endometrial carcinomas” and any possible precursors; (2) none of the 131 patients had past history of hormone therapy which may obscure the natural history of endometrial carcinomas [5]; and (3) our study was based on the large number of the patients (131 cases) with “small endometrial carcinoma.”

## Material and Methods

The present prospective study of “small endometrial carcinoma” began in January 1986 and was closed to all patient entry in December 2000.

During the period 1986–2000, 940 women with endometrial carcinoma were treated by surgery at The Cancer Institute Hospital of Japan. They underwent preoperative endometrial biopsy for diagnosis. Neither preoperative radiotherapy nor chemotherapy was employed in these cases.

An effort was made to evaluate the size of endometrial carcinomas macroscopically using the removed fresh uteri. In all cases with endometrial carcinomas less than 10 mm in the largest dimension macroscopically, the fixed uterine corpus was step-cut with each piece being 3–4 mm in thickness for embedding. The number of sections of the corpus ranged from 19 to 48 with mean of 27. The tumor size evaluated macroscopically was reconfirmed microscopically. Microscopy was used and more precise measurements were made in situations where the lesions were diffusely abnormal macroscopically and difficult to assess macroscopically.

An epidemiologic questionnaire comprised 30 questions pertaining to age, the initial manifestation, menstrual and obstetric history, use of hormones, general medical history, and family history. The questionnaire was completed by the patient at her first visit and subsequently reviewed with her by well-trained and experienced interviewers who clarified any questions not understood by the patient. Weight, height, and blood pressure of the patient were measured at her admission and recorded by the nurses. Associated medical conditions including obesity, diabetes mellitus, and hypertension were recorded. For this purpose the hypertension was defined as a blood pressure of greater than 150 systolic and/or greater than 90 diastolic. Obesity was defined as body mass index (wt., kg/ht, m^2^) of 25 or more which World Health Organization (WHO) recommended to reduce weight. A diagnosis of diabetes mellitus was based on WHO's recommendation. A history of previous estrogen exposure or pelvic irradiation was also recorded. Estrogen exposure was defined as a record of at least 6 months of estrogen usage. Menopause was defined as no bleeding for 12 months as a result of a depletion of ovarian follicles. Late menopause was defined as onset of menopause past the age of 53 years which was related to an increased risk of endometrial carcinomas [6].

There were 131 patients with “small endometrial carcinoma.” The present study was based on these cases. All of the patients were Japanese. Forty-five cases underwent dilatation and curettage (D and C) after endometrial biopsy because endometrial biopsy revealed suspicious diagnosis of malignancy. There was a small amount of carcinoma measuring less than 5 × 5 mm in hematoxylin and eosin–stained sections of D and C specimens. No difference in the amount of carcinoma between office biopsy specimens and D and C specimens was noted. Therefore difference of pathologic diagnosis procedures, office biopsy or D and C, did not contribute to difference of the size of endometrial carcinoma in the uteri removed. Informed consent was obtained from the patients. The types of surgery for them included total abdominal hysterectomy and bilateral salpingo-oophorectomy with or without lymphadenectomy. Sixty-nine women underwent pelvic lymphadenectomy and 45 women underwent both pelvic and aortic lymphadenectomy. The number of lymph nodes examined in the former ranged from 18 to 40 (mean, 28.3) and that in the latter ranged from 30 to 70 (mean, 50.2). Neither postoperative adjuvant chemotherapy nor radiotherapy was used for them.

Potential controls were 709 women who underwent operation (total abdominal hysterectomy and bilateral salpingo-oophorectomy with or without lymphadenectomy) between the periods of 1986–2000 and had endometrial carcinoma more than 10 mm in diameter. The pool of potential controls was reduced to 262 by the selection at random of two controls per cases from the same calendar year. Two hundred and fifty-one controls underwent pelvic lymphadenectomy with or without aortic lymphadenectomy. The number of lymph nodes examined in the former ranged from 16 to 51 (mean, 29.2) and that in later ranged from 24 to 95 (mean, 51.3).

Endometrial carcinomas were categorized using classification recommend by WHO and graded according to the 1988 modified International Federation of Gynecology and Obstetrics (FIGO) system [7]. Tumors composed of a mixture of endometrioid adenocarcinoma and serous adenocarcinoma were classified serous adenocarcinoma if more than 25% of the tumor was serous adenocarcinoma, because Sherman et al. [8] have reported that such tumor behaves as pure serous adenocarcinoma. Endometrial hyperplasia was classified as simple, complex, or atypical hyperplasia using classification recommended by WHO [4].

Hematoxylin and eosin–stained sections of the hysterectomy and curettage specimens were reviewed. In each case the following histopathologic data were observed and recorded: histologic type of cancer, grade, presence or absence of endometrial hyperplasia, and depth of myometrial invasion. The presence or absence of endometrial intraepithelial carcinoma described by Ambros et al. [9] was recorded.

The histopathologic differential diagnosis of well-differentiated endometrioid adenocarcinoma from hyperplasia was made when atypical endometrial glands invaded their own stroma. In this study, we accepted Silverberg's criteria for the presence of stromal invasion: (1) total absence of stroma between glands, (2) fibrosis of stroma between glands, and (3) necrosis of stroma between glands [4].

The Kaplan–Meier method was used to estimate overall survival (OS) and progression-free survival (PFS). The log-rank test was used to assess difference in survival between groups of patients. Quantitative variables (age at operation, age at onset of menopause, and menarche) were expressed as mean and ranges and compared with Werch's *t*-test. Categorical variables were compared with the chi-squared or Fisher's exact test. A value of *P* < 0.05 was considered statistically significant.

## Results

### Incidence

There were 131 patients with “small endometrial carcinoma” among 940 (13.9%) patients who underwent operation for endometrial cancer.

### Histopathologic features

**Figure 1:**
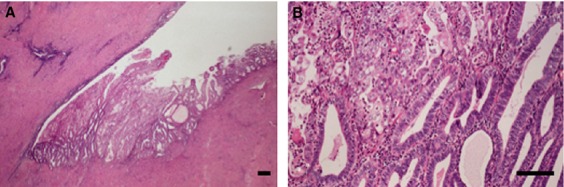
(A) A section of the uterine fundus showing “small endometrial carcinoma” of elevated type arising from the tubal recess (cornu) with associated endometrial complex hyperplasia adjacent to the cancer (H&E, original magnification 40×). Scale bar = 100 μm. (B) Higher magnification of the field illustrated in (A) showing endometrioid adenocarcinoma, grade 2 (left) and endometrial complex hyperplasia without atypia (right) (H&E, original magnification 200×). Scale bar = 100 μm.

**Figure 2:**
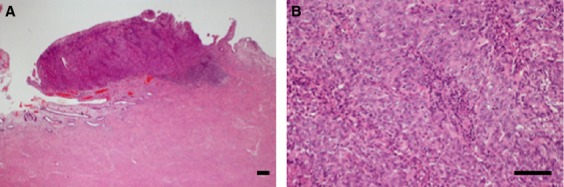
(A) A section of the uterine corpus showing “small endometrial carcinoma” of elevated type not associated with endometrial hyperplasia (H&E, original magnification 40×). Scale bar = 100 μm. (B) Higher magnification of the field illustrated in (A) showing endometrioid adenocarcinoma, grade 3 (H&E, original magnification 200×). Scale bar = 100 μm.

**Figure 3:**
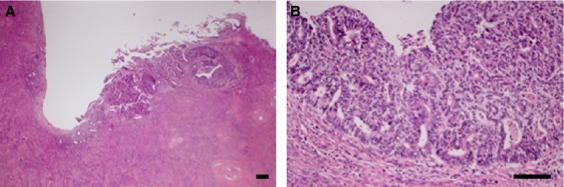
(A) A section of the uterine corpus showing “small endometrial carcinoma” of flat type not associated with endometrial hyperplasia (H&E, original magnification 40×). Scale bar = 100 μm. (B) Higher magnification of the field illustrated in (A) showing endometrioid adenocarcinoma, grade 2 (H&E, original magnification 200×). Scale bar = 100 μm.

As shown in Table 1, the 131 patients were classified into two groups according to the status of the uninvolved endometrium adjacent to the cancer. Among 131 cases, 53 were associated with hyperplasia of the endometrium adjacent to “small endometrial carcinoma” (Group I) and 78 were not associated with hyperplasia (Group II). The associated hyperplasia in Group I was exclusively of complex type with or without atypia (Fig. 1). Associated atypical complex hyperplasia was found in 22 cases. The endometrium in Group II was normally cycling or atrophic (Figs. 2 and 3). Histologic types of “small endometrial carcinomas” and the controls were shown in Table 2. The histologic types of “small endometrial carcinomas” in Groups I and II were shown in Table 3. Of 53 tumors in Group I, 52 (98.1%) were grades 1 and 2 endometrioid adenocarcinoma, none (0%) were grade 3 endometrioid adenocarcinoma, and one (1.9%) was adenosquamous carcinoma. Of 78 carcinomas in Group II, 56 (71.8%) were grades 1 and 2 endometrioid adenocarcinoma, 6 (7.7%) were grade 3 endometrioid adenocarcinoma, and 15 (19.2%) were nonendometrioid carcinoma (13 serous and 2 clear cell adenocarcinomas). There was statistical difference in the frequency of grades 1 and 2 endometrioid adenocarcinoma between Groups I and II (52/53 vs. 56/78, *P* = 0.00026). All of grade 3 endometrioid, serous, and clear cell adenocarcinomas were exclusively in Group II. Among 108 grades 1 and 2 endometrioid adenocarcinomas which were traditionally considered to be associated with endometrial hyperplasia, 52 (48.1%) cases were associated with hyperplasia (Group I) and 56 (51.8%) cases were not associated with hyperplasia (Group II). Four (30.8%) in 13 serous adenocarcinomas were associated with endometrial intraepithelial carcinoma whereas none of endometrioid carcinomas or clear cell adenocarcinoma was associated with it.

**Table 1:** Status of the tumor-free endometrium adjacent to “small endometrial carcinomas.”

Status of the tumor-free endometrium	Number of cases (%)
Hyperplastic endometrium (Group I)	53 (40)
Complex hyperplasia only	31
Atypical hyperplasia only	0
Both complex and atypical hyperplasia	22
Simple hyperplasia only	0
Nonhyperplastic endometrium (Group II)	78 (60)
Total	131 (100)

**Table 2:** Histologic types of “small endometrial carcinomas” and controls

	“Small endometrial carcinomas” (*n*=131)	Controls (*n*=262)	*P*-value
Endometrioid carcinoma			
Endometrioid adenocarcinoma			
Grades 1 and 2	108 (82.4%)	191 (72.9%)	0.0012
Grade 3	6 (4.6%)	21 (8.0%)	0.34
Adenoacanthoma	1 (0.8%)	10 (3.8%)	0.18
Adenosquamous carcinoma	1 (0.8%)	20 (7.6%)	0.011
Subtotal	116 (88.6%)	242 (92.3%)	
Nonendometrioid carcinoma			
Serous papillary adenocarcinoma	13 (9.9%)	6 (2.3%)	0.0028
Clear cell adenocarcinoma	2 (1.5%)	4 (1.5%)	0.95
Squamous cell carcinoma	0 (0%)	0 (0%)	–
Mucinous adenocarcinoma	0 (0%)	2 (0.8%)	0.60
Mixed carcinoma	0 (0%)	8 (3.1%)	0.017
Undifferentiated carcinoma	0 (0%)	0 (0%)	–
Subtotal	15 (11.4%)	20 (7.7%)	

**Table 3:** Histologic types of “small endometrial carcinomas” in the Groups I (with associated hyperplasia) and II (without associated hyperplasia)

	Group I (*n*=53)	Group II (*n*=78)	*P*-value
Endometrioid carcinoma			
Endometrioid adenocarcinoma			
Grades 1 and 2	52 (98.1%)	56 (71.8%)	0.00026
Grade 3	0 (0%)	6 (7.7%)	0.10
Adenoacanthoma	0 (0%)	1 (1.3%)	1.00
Adenosquamous carcinoma	1 (1.9%)	0 (0%)	0.85
Subtotal	53 (100%)	63 (80.8%)	
Nonendometrioid carcinoma			
Serous papillary adenocarcinoma	0 (0%)	13 (16.7%)	0.0050
Clear cell adenocarcinoma	0 (0%)	2 (2.5%)	0.65
Squamous cell carcinoma	0 (0%)	0 (0%)	–
Mucinous adenocarcinoma	0 (0%)	0 (0%)	–
Mixed carcinoma	0 (0%)	0 (0%)	–
Undifferentiated carcinoma	0 (0%)	0 (0%)	–
Subtotal	0 (0%)	15 (19.2%)	

### Clinical features and biological behavior

History of estrogen use was not noted and no family history of Lynch syndrome in any of the 131 patients with “small endometrial carcinoma” was noted.

As shown in Table 4, the age of 131 patients with “small endometrial carcinoma” at the time of operation ranged from 27 to 75 years and the mean age was 54.0 years. They were significantly younger than the control patients with endometrial carcinoma more than 10 mm (mean 54.0 vs. 58.9; *P* < 0.0001). The predominance of “small endometrial carcinomas” was in the age group 50–54 years old and that of the controls was in the age group of 55–59. The age distribution of “small carcinoma” corresponded with that of controls with exception of the age group 45–49 and 55–59. “Small carcinomas” showed sharp increase in the age group 45–49 and sharp decrease in the age group 55–59.

**Table 4:** Age distribution of cases with “small endometrial carcinomas” and controls

	Cases with “small endometrial carcinomas” (*n*=131)	Controls (*n*=262)	*P*-value
Age at diagnosis (years)			
Mean	54.0	58.9	<0.0001
Range	27–75	32–80	
Age distribution (%)			
≤39years	5.3	0.5	
40–44years	4.6	2.7	
45–49years	16.0	10.0	
50–54years	32.1	21.6	
55–59years	13.7	22.4	
60–64years	14.5	21.6	
65–69years	9.2	11.6	
70–74years	3.8	5.6	
75≤years	0.8	3.8	

As shown in Table 5, 27 (20.6%) of the 131 patients with “small endometrial carcinoma” and 12 (4.6%) of the controls were asymptomatic. Chi-squared test showed significant difference between the two groups (*P* < 0.0001). Among 131 patients with “small endometrial carcinomas” myometrial invasion was noted in 36 (27.5%) patients. Myometrial invasion was found in 259 (98.9%) among 262 controls. There was significant difference between the cases and controls (*P* < 0.0001). There was no adnexal metastasis in the patients with “small endometrial carcinoma” except for a patient with endometrioid grade 1 adenocarcinoma (0.8%). Adnexal metastasis was found in seven controls (2.7%). Among 114 patients with “small endometrial carcinoma” who underwent pelvic lymphadenectomy with or without aortic lymphadenectomy, nodal metastasis was present in a patient with endometrioid grade 1 adenocarcinoma (0.9%). Among 251 controls that underwent pelvic lymphadenectomy with or without aortic lymphadenectomy nodal metastasis was noted in 33 (13.1%). The difference was statistically significant (*P* = 0.00039).

**Table 5:** Clinicopathologic features of cases with “small endometrial carcinomas” and controls

	Number of cases with “small endometrial carcinoma” (*n*=131)	Number of controls (*n*=262)	*P*-value
Asymptomatic patients	27 (20.6%)	12 (4.6%)	<0.0001
Myometrial invasion	36 (27.5%)	259 (98.9%)	<0.0001
Ovarian metastasis	1 (0.8%)	7 (2.7%)	0.38
Lymphatic metastasis	1 (0.9%)^1^	33 (13.1%)^1^	0.00039

1 Percentage in the patients who underwent lymphadenectomy.

A summary of clinical features of the “small endometrial carcinoma” patients in Groups I (with associated hyperplasia) and II (without associated hyperplasia) is presented in Table 6. The mean age of the patients in Group I was 51.8 years (27–74) and that in Group II was 55.5 years (31–75). The patients in Group I were significantly younger than those in Group II (*P* = 0.026). More of the patients in Group II were postmenopausal than were those in Group I (62.8% vs. 26.4%, *P* < 0.0001). There was no significant difference between the two groups in mean age of menarche (*P* = 0.75). The frequencies of constitutional risk factors of endometrial carcinoma (late menopause, obesity, hypertension, diabetes, nulligravidity, nulliparity) for the two groups were shown in Table 6. There was no statistical difference in the frequencies of late menopause, obesity, hypertension, diabetes, nulligravidity, and nulliparity between Groups I and II (*P* > 0.05).

**Table 6:** Clinical and demographic features of “small endometrial carcinoma” in the Groups I (with associated hyperplasia) and II (without associated hyperplasia)

	Total (*n*=131)	Group I (*n*=53)	Group II (*n*=78)	*P*-value
Age at diagnosis (range), years	54.0 (27–75)	51.8 (27–74)	55.5 (31–75)	0.026
Postmenopausal, case	63 (48.2%)	14 (26.4%)	49 (62.8%)	<0.0001
Age at menarche (range), years	13.7 (10–21)	13.8 (10–18)	13.7 (11–21)	0.75
Age at menopause (range), years	51.5 (38–58)	51.7 (42–58)	51.4 (38–58)	0.61
Late menopause^1^, case	21 (33.3%)^2^	6 (42.9%)^2^	15 (30.6%)^2^	0.59
Obesity^3^, case	15 (11.5%)	8 (15.1%)	7 (9.0%)	0.42
Hypertension, case	18 (13.7%)	10 (18.9%)	8 (10.3%)	0.25
Diabetes, case	3 (2.3%)	0 (0%)	3 (3.8%)	0.39
Nulligravida, case	20 (15.3%)	11 (20.8%)	9 (11.5%)	0.23
Nulliparity, case	26 (19.8%)	14 (26.4%)	12 (15.4%)	0.18

1 Onset of menopause past the age of 53years.

2 Persentage in postmenopausal patients.

3 Body mass index of 25 or more.

### Macroscopic types and location

Of 131 patients, 107 (81.7%) had a single lesion and 24 (18.3%) had multiple lesions. Macroscopically “small endometrial carcinomas” in 74 (56.5%) patients were elevated (Figs. 1A and 2A) and those in 57 (43.5%) were flat (Fig. 3A). None of the patients had depressed lesions (Table 7A). When the uterine corpus was divided into three portions – the upper, middle, and lower third sections, “small endometrial carcinomas” in 87 (66.4%) patients were located in the upper section, those in 28 (21.4%) patients in the middle, those in 4 (3.0%) patients in the lower, and those in 12 (9.2%) patients in both upper and middle sections (Table 7B). Ninety-nine (75.6%) patients had “small endometrial carcinomas” located in the upper third section and among them 32 (32.2%) had the carcinomas in the cornu (Fig. 1A).

**Table 7:** Macroscopic types and location of “small endometrial carcinomas” (*n*=131)

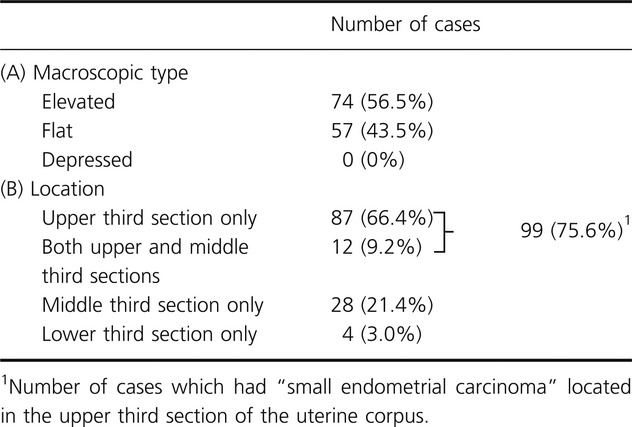

### Prognosis

All the 131 patients were followed at least 5 years. Five-year OS and PFS of patients with “small endometrial carcinoma” was 0.962 and 0.916, respectively.

As shown in Table 8, 5-year OS and PFS by histological type were endometrioid carcinoma 0.983 and 0.983; nonendometrioid carcinoma 0.800 and 0.733. Patients with endometrioid carcinoma had significantly better 5-year OS (*P* < 0.001) and PFS (*P* < 0.001) compared to patients with nonendometrioid carcinoma.

**Table 8:** Prognosis of patients with “small endometrial carcinoma” by histological types

	Endometrioid carcinoma (*n*=116)	Nonendometrioid carcinoma (*n*=15)	*P*-value
Five-year OS (95% CI)	0.983 (0.959–1.000)	0.800 (0.621–1.000)	<0.001
Five-year PFS (95% CI)	0.983 (0.959–1.000)	0.733 (0.540–0.995)	<0.001

CI, confidence interval; OS, overall survival; PFS, progression-free survival.

As show in Table 9, 5-year OS and PFS by the two groups of “small endometrial carcinoma” were Group I (with associated hyperplasia) 0.962 and 0.962; Group II (without associated hyperplasia) 0.962 and 0.949. There was no significant difference in 5-year OS (*P* = 0.97) and PFS (*P* = 0.72) between the two groups.

**Table 9:** Prognosis of patients with “small endometrial carcinoma” in the Groups I (with associated hyperplasia) and II (without associated hyperplasia)

	Group I (*n*=53)	Group II (*n*=78)	*P*-value
Five-year OS (95% CI)	0.962 (0.912–1.000)	0.962 (0.920–1.000)	0.970
Five-year PFS (95% CI)	0.962 (0.912–1.000)	0.949 (0.901–0.999)	0.720

CI, confidence interval; OS, overall survival; PFS, progression-free survival.

## Discussion

The knowledge of the biologic behavior and clinicopathological features of smaller carcinomas is important for early diagnosis and treatment [10, 11]. However, in reference to endometrial carcinoma it is not clear, because reported cases of smaller carcinomas were of limited number. Using the methods of serial block sections of the entire endometrium of the uterus surgically removed for smaller endometrial carcinomas, we found 131 “small endometrial carcinomas.” Patients with “small endometrial carcinoma” were, on average, 5 years younger than were those whose tumors measured more than 10 mm in diameter. “Small endometrial carcinomas” showed sharp increase in the age group 45–49 years and were predominant in that of 50–54.

The majority of the lesions were single and developed in the upper third of the corpus. Recognition of this fact is very important not to miss “small endometrial carcinomas” by curettage or hysteroscopic examination. Approximately a half of “small endometrial carcinomas” were of flat type which were very difficult to find macroscopically even in the surgically resected uteri. Thus it may be difficult to find “small endometrial carcinoma” of flat type by hysteroscopy or transvaginal ultrasound.

Incidence of nodal and adnexal metastases was low (<1%) in patients with “small endometrial carcinomas.” Prognosis of the patients with “small endometrial carcinoma” is favorable (5-year PFS: 0.96). Prognostically “small endometrial carcinomas” are divided into the two entities, endometrioid type and nonendometrioid type. Prognosis of endometrioid type is favorable (5-year PFS: 0.98), but even in “small endometrial carcinomas” nonendometrioid type has unfavorable prognosis (5-year PFS: 0.73). The difference is statistically significant (*P* < 0.001). Tumor size alone cannot be used as the only defining criteria for highly curable endometrial carcinomas.

The means of histopathological sampling best suitable to early detection is still debatable. The big disadvantage for the office biopsy is that the material is only a sample of the entire endometrium. Our present study indicated that the majority of the patients (75.6%) had “small endometrial carcinomas” located in the upper thirds of the uterine corpus. Especially it is important to recognize that many “small endometrial carcinomas” (32 cases) were found in the tubal recesses (cornu). Every gynecologist has had the experience of missing a carcinoma lurking in a cornu. When the slightest suspicion of endometrial carcinoma exists, the entire endometrial cavity, especially the upper third section of the corpus, should be carefully sampled for early detection.

It is generally agreed that an increase in early diagnosis may lead to an increasing proportion of asymptomatic patients. Actually in our study, 20.6% of patients with “small endometrial carcinoma” were asymptomatic although only 4.6% of the patients with tumor more than 10 mm (control) were asymptomatic (*P* < 0.0001).

Histopathologic study of endometrial small-sized carcinomas and their adjacent noncancerous endometrium enables us to speculate the probable histogenesis of endometrial carcinoma, because small carcinomas are in the incipient phase and it seems logical to assume that they arise from its adjacent endometrium. Our study of “small endometrial carcinoma” demonstrates that two distinct mechanisms may be possible for onset of endometrial carcinoma: endometrial carcinoma occurring from endometrial complex hyperplasia and endometrial carcinoma occurring ab initio from normal endometrium (de novo carcinoma). The hypothesis that there may be two pathways of endometrial carcinogenesis proposed by many authors [1, 2] was confirmed by our present study of “small endometrial carcinomas.”

Many studies implicated complex hyperplasia in the development of endometrial carcinoma. If such were the case, most of endometrial carcinomas could be prevented by early detection and treatment of endometrial hyperplasia [3]. Our present study of “small endometrial carcinomas” free of the biasing factors mentioned in “Introduction” of this paper, which increase or decrease the actual frequency strongly suggests approximately 60% of endometrial carcinomas occur ab initio. Considering the high incidence of endometrial de novo carcinomas, changes in strategies for detection and prevention of endometrial carcinoma are urgent. Possibly we cannot prevent advanced endometrial carcinoma only by detection and treatment of hyperplasia. Much effort must be focused on detecting small de novo carcinomas.

Is there any difference in risk factors between Groups I (with associated hyperplasia) and II (without associated hyperplasia)? Several studies have evaluated risk factors separately for the two groups, but most reports were not reliable because the number of subject being evaluated were small, an approach to recording clinical information was not uniform, definition of each risk factors was not clear, and finally statistic evaluation was not valid. Preliminary study by Westhoff et al. [12] showed earlier menarche and higher weight might be risk factors in Group I, but the more precise epidemiologic study by Sivrids et al. [13] reported that the demographic features of the two groups were similar. Our present study has shown that there is no difference in risk factors between the two groups at early stage of a multistage process of endometrial carcinogenesis.

Is there any justification to subdivide endometrial carcinoma into Groups I (with associated hyperplasia) and II (without associated hyperplasia) based on the pathological features of nonneoplastic endometrium? Sivridis et al. [13] has revealed that a difference in prognosis between the two groups is due to the higher incidence of grades 2 and 3 carcinomas in Group II. There was no significant difference in prognosis of grade 1 carcinomas between the two groups. Thus, the presence or absence of complex hyperplasia is probably not an independent prognostic factor. This subdivision has no therapeutic implication although it is possibly important pathogenetically. Studies of molecular mechanisms of endometrial carcinogenesis may be more precise and accurate if Groups I and II are considered. We previously identified one set of genes differentially expressed in Groups I and II “small endometrial carcinoma” [14].

Are there any possible precursors of endometrial carcinoma, which lacks a background of hyperplasia? Ambros et al. [9] and Spiegel [15] identified endometrial intraepithelial carcinoma in a high proportion of uteri containing invasive endometrial serous adenocarcinomas without associated hyperplasia. They have proposed that endometrial intraepithelial carcinoma is an in situ precursor of serous adenocarcinomas. We found endometrial intraepithelial carcinoma in 30.8% (4/13) of “small endometrial carcinomas” of serous type but it was not noted in “small endometrial carcinomas” of endometrioid type. Thus, it is reasonable to presume that endometrial intraepithelial carcinoma may be an in situ precursor of serous adenocarcinoma but it is not a precursor of endometrioid carcinoma.

**Figure 4:**
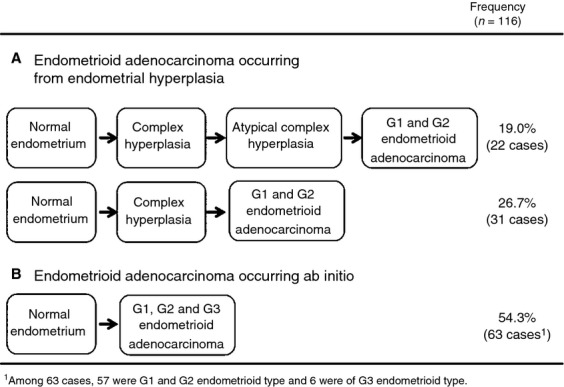
Diagram of development of endometrioid adenocarcinoma based on our study of 116 cases of “small endometrial carcinoma of endometrioid type.” (A) Endometrioid adenocarcinoma occurring from endometrial hyperplasia. (B) Endometrioid adenocarcinoma occurring ab initio.

Figure 4 is a diagram of development of endometrioid adenocarcinoma from normal endometrium based on our present study. As shown in Figure 4, 19.0% of “small endometrial carcinomas of endometrioid type” developed from complex atypical hyperplasia, 26.7% arose from complex hyperplasia without an intervening phase of complex atypical hyperplasia, and 54.3% occurred ab initio (de novo carcinoma). It is important to recognize that almost half cases (49.1%) of grades 1 and 2 endometrioid adenocarcinoma occurred ab initio.

What is the treatment of choice for control of “small endometrial carcinoma”? As the risk of lymphatic metastasis was low (less than 1.0%) and no recurrence was found in patients with “small endometrial carcinoma” of endometrioid type, lymphadenectomy can be discarded and total hysterectomy and bilateral salpingo-oophorectomy may be the treatment of choice for them.

The value of tumor size assessed intraoperatively in disease management has been demonstrated in endometrial carcinoma. Mayo Clinic group has disclosed that endometrioid histology and tumor size is useful to determine extent of surgery [16]. They have also shown that macroscopic assessment of tumor size correlated with final pathology. As patients with grades 1 or 2 endometrioid carcinoma and tumor dimension <2 cm measured intraoperatively have a low probability of lymphatic spread Mayo Clinic group suggests that lymph adenectomy can be omitted [16].
